# Impact of instrumentation techniques on post-operative pain in endodontics

**DOI:** 10.6026/973206300210011

**Published:** 2025-01-31

**Authors:** Saif Hadi Alyami

**Affiliations:** 1 Department of Restorative Dentistry, College of Dentistry, Najran University, Najran, Saudi Arabia

**Keywords:** Manual instrumentation, postoperative pain, root canal preparation, rotary instrumentation

## Abstract

The effect of manual and rotary instrumentation on postoperative pain in teeth with asymptomatic irreversible pulpitis is of interest. Hence, we used a sample from 100 subjects (50 mandibular molars per group). Participants underwent endodontic treatment using either manual or rotary techniques. Pain severity was assessed at multiple intervals. Results indicated a significant reduction in postoperative pain in both groups with no statistically significant differences between them. Rotary instrumentation demonstrated numerically lower pain levels but without statistical significance.

## Background:

The incidence of postoperative pain after endodontic treatment has been reported to be around 30.8% [1]. Several factors are implicated in the development of pain and discomfort after root canal procedures, including insufficient instrumentation, the unintended expulsion of irrigation solutions, the release of intracanal dressings, traumatic occlusion, overlooked canals, pre-existing pain, periapical pathosis and the expulsion of apical debris [2]. Research indicates that the apical extrusion of infected debris during chemo-mechanical instrumentation is a primary cause of periapical inflammation and subsequent postoperative pain [3]. Various elements influence the extrusion of debris, such as the irrigation protocol, the final apical size, the duration of root canal instrumentation, the techniques utilized and the design of the instruments [4]. All instrumentation methods lead to some degree of apical debris extrusion, regardless of the precautions taken to limit preparation to the apical terminus [5, 6-7]. Nonetheless, some rotary techniques are reported to reduce debris extrusion more effectively than others. Therefore, it is of interest to compare manual and rotary instrumentation regarding their impact on postoperative pain in teeth with asymptomatic irreversible pulpitis.

## Materials and Methods:

With 50 mandibular molars in each group, the sample size was 100 subjects. Participants were selected based on their need for endodontic treatment due to asymptomatic irreversible pulpitis affecting either their mandibular first or second molars, all of which exhibited normal periapical radiographic findings. The periapical radiographs were meticulously processed and archived utilizing a specialized scanner and software interface before undergoing further analysis with Rinn XCP devices and a digital radiography system. Following the selection of subjects, a physician organized the 100 participants into two distinct groups, each consisting of 50 individuals. The groups were matched in terms of gender and the distribution of mandibular first and second molars, specifically those with three and four root canals.

## Results:

In both manual and rotary groups, there was a significant reduction in postoperative pain severity from the initial assessment to the final evaluation across all time points measured (P<0.001). Nevertheless, a comparative analysis of pain severity between the RaCe rotary and hand K-Flexofile groups did not indicate any statistically significant differences (P=0.79). Specifically, the mean pain severity scores recorded four hours post-treatment were 27.14 ± 5.32 for the RaCe group and 35.45 ± 6.72 for the K-Flexofile group. After eight hours, the scores were 25.11±4.78 for the rotary group and 30.63±3.84 for the hand file group. Although the rotary group exhibited lower pain severity at both time intervals compared to the hand file group, the differences were not statistically significant (P>0.05). Furthermore, at the twelve-hour, twenty-four-hour, forty-eight-hour and seven-day intervals, the differences in pain severity between the two groups remained insignificant (P>0.05) (Table 1).

## Discussion:

Post-endodontic pain represents a significant challenge for patients, adversely affecting the relationship between the patient and clinician [8]. Despite substantial advancements in both tools and pharmacological treatments, the occurrence of pain following endodontic procedures continues to be a prevalent issue [9, 10-11], with reported frequencies varying between 1.9% and 48% in existing studies. This wide variability is likely attributable to differences in research methodologies and the criteria used to define post-operative pain [12]. Even when optimal protocols are adhered to, the literature indicates that mild post-endodontic pain occurs with a frequency of 10-30%, while severe pain is reported at rates of 6-12% [13, 14, 15- 16]. Numerous etiological factors contribute to postoperative pain, including a history of preoperative discomfort, inadequate canal debridement, hyper-occlusion, periapical pathology and the extrusion of debris into the periapical region [17]. The extrusion of infected dentin into the periapical tissue has been identified as a significant contributor to pain following endodontic procedures. While the extrusion of debris is a common occurrence, even when instrumentation is confined to the canal, various instruments appear to be linked to differing levels of debris extrusion. Some research indicates that hand files may result in greater amounts of extruded debris compared to engine-driven files, attributed to the Archimedes screw effect associated with full rotational movement. Bürklein *et al.* demonstrated that a single-file reciprocating system (Reciproc) resulted in more debris extrusion than two single-file rotary systems (OnShape and F360) [18, 19-20].

This study found no significant difference in postoperative pain between manual and rotary instrumentation (P>0.05), aligning with Talebzadeh *et al.* who reported no pain severity differences between RaCe rotary systems and hand K-Flexofiles [21]. Similarly, Shahi *et al.* observed no significant pain differences between RaCe and Pro-Taper rotary instruments [22]. However, other studies indicate that K-files may cause more pain than rotary systems [23]. Arias *et al.* noted longer-lasting pain with rotary preparation [24], while Kashefinejad *et al.* found significantly fewer analgesic needs in the rotary group (13.3%) compared to the K-file group (56.7%) [25]. Similarly, Shandilya *et al.* reported lower pain severity with ProTaper Next rotary instruments compared to K-files [26, 27-28] and Makanjuola *et al.* highlighted the long-term benefits of rotary techniques, including reduced periapical radiolucency and better outcomes [29. These findings suggest that the choice of instrumentation method influences postoperative pain and treatment success. The strengths of this study include sample size, controlled gender and molar type and consistent measurement of postoperative pain at multiple time intervals. However, a limitation is the lack of long-term follow-up beyond one week, which may not fully capture the prolonged effects of different instrumentation techniques on postoperative pain.

## Conclusion:

Manual and rotary instrumentation both effectively reduce postoperative pain with no significant difference between them. Rotary techniques may provide a slight advantage. However, addition data is needed to confirm these observations.

## Figures and Tables

**Table 1:** Pain severities in 2 groups based on VAS

**Interval**	**Rotary**	**Manual**
4 hours	27.14±5.32	35.45±6.72
8 hours	25.11±4.78	30.63±3.84
12 hours	18.16±3.46	29.45±3.03
24 hours	14.84±3.12	15.66±3.16
48 hours	8.85±1.13	9.30±1.17
1 week	2.65±1.76	2.98±1.53
